# Expression and Clinical Significance of Cancer Stem Cell Markers CD24, CD44, and CD133 in Pancreatic Ductal Adenocarcinoma and Chronic Pancreatitis

**DOI:** 10.1155/2017/3276806

**Published:** 2017-06-04

**Authors:** L. Durko, W. Wlodarski, O. Stasikowska-Kanicka, M. Wagrowska-Danilewicz, M. Danilewicz, P. Hogendorf, J. Strzelczyk, E. Malecka-Panas

**Affiliations:** ^1^Department of Digestive Tract Diseases, Medical University of Lodz, Lodz, Poland; ^2^Department of Pathomorphology, Medical University of Lodz, Lodz, Poland; ^3^Department of General and Transplant Surgery, Medical University of Lodz, Lodz, Poland

## Abstract

Cancer stem cells (CSC) play an important role in pancreatic carcinogenesis and prognosis. The study aimed at examining the expression of CD24, CD44, and CD133 in human PDAC and CP in order to evaluate its clinicopathological correlations and the clinical significance. Surgical specimens from 23 patients with PDAC and 15 patients with chronic pancreatitis after pancreatic resection were stained with CD24, CD44, and CD133 antibodies. The intensity of staining was scored from 0 (negative) to 3 (strongly positive). *Results*. Mean CD24 staining score in PDAC was 1.38 ± 0.76 and was significantly higher than that in CP: 0.70 ± 0.53 (*p* < 0.01); CD44 score in PDAC was 2.23 ± 0.42 and was significantly higher than that in CP: 1.87 ± 0.55 (*p* < 0.05); CD133 score 0.93 ± 0.58 was not different from CP: 0.71 ± 0.43 (*p* > 0.05). CD44 immunoreactivity was significantly higher (*p* < 0.05) in pT1 and pT2 patients together as regards pT3: 2.45 ± 0.37 versus 2.06 ± 0.38 as well as in N0 patients compared to N1 patients: 2.5 ± 0.38 versus 2.04 ± 0.34. *Conclusions*. CD24 and CD44 are upregulated in human pancreatic cancer compared to chronic pancreatitis. CD44 immunoreactivity decreases with the tumor advancement and may represent the negative PDAC prognostic factor. Each CSC marker was differently related to PDAC advancement. CD133 may lack clinical significance in PDAC.

## 1. Introduction

The incidence of and mortality associated with pancreatic ductal adenocarcinoma (PDAC) have increased during the last decade. It is expected that by the year 2030, pancreatic cancer will become the second most prevalent cause of cancer-related deaths [1]. One of the reasons associated with high malignancy of PDAC is the presence of a subpopulation of chemoresistant, self-renewable, and multipotent cells in the bulk of tumor termed cancer stem cells (CSC). These cells are believed to be responsible for tumor initiation, rapid growth, resistance to therapy, recurrence, and metastasis. CSC have been found in several types of neoplasms [2–5] including pancreatic cancer [6, 7]. In 2007, Li and colleagues identified a highly tumorigenic subpopulation of cells in human PDAC. Transplantation of this subpopulation of cells to immunocompromised mice led to initiation and systemic spread of pancreatic cancer in experimental animals. Interestingly, these cells express surface markers CD44, CD24, and epithelial-specific antigen (ESA). Such phenotype was present in 0.2–0.8% of PDAC cells and corresponded to their 100-fold increased tumorigenic potential compared with nontumorogenic cancer cells [6].

Hermann et al. demonstrated that pancreatic CSC show high expression of CD133 (prominin-1) [7]. CD133 is a transmembrane glycoprotein responsible for regulation of multiple cell signalling pathways, including Akt/PKB, Bcl-2, Ras, and its downstream effectors such as ERK, JNK, PI3K, and p38K [8].

CD24, a small cell surface protein anchored by glycosylphosphatidylinositol, is heavily glucosylated and is involved in cell-cell and cell-matrix interactions. CD24 tends to be expressed at higher levels in progenitor cells and metabolically active cells and to a lesser extent in well-differentiated cells. The function of CD24 is unclear for most types of cells [9].

CD44 is the transmembrane glycoprotein that can act as a receptor for extracellular matrices as hyaluronic acid and is the downstream target of the Wnt/*β*-catenin pathway. CD44 expression was related to a more aggressive course and presence of metastases in PDAC [10, 11].

The CSC presenting self-renewable properties involve also other signaling pathways such as Notch and Hedgehog. Overexpression of Notch-1 results in increased cell proliferation, migration, and invasion of cancer cell lines. Additionally, high levels of CD44 were observed in PDAC with increased Notch-1 expression [12].

Pancreatic CSC also influence the Hedgehog signaling pathway. It has been shown that a ninefold increase in Sonic hedgehog mRNA levels were present in the CD44 and CD24 PDAC positive cells compared to the pancreatic cancer cells with low or absent expression of these biomarkers [6]. Interestingly, Rodova et al. showed that blocking hedgehog pathway by sulforane inhibits the proliferation of CSC [13].

Another mechanism involved in CSC function involves mTOR pathway. The mTOR inhibition by rapamycin in in vitro studies resulted in decreased viability of CD133 PDAC cells and their reduced self-renewal properties [14].

Many authors have proven that isolated pancreatic CSC are more chemoresistant compared to non-CSC cancer cells [15–17]. Therefore, elimination of CSC in PDAC could improve the treatment results and lead to a better prognosis in patients affected by this disease. Multiple new strategies to target stem cells are being investigated [18–20]. The more precise identification of pancreatic CSC may help to better understand the biology of PDAC and allow for successful development of more accurate and targeted therapies.

Chronic pancreatitis (CP) is associated with increased risk of PDAC [21]. Nevertheless, the nature of the transition between CP and PDAC is poorly understood. Fibrosis is a hallmark of human PDAC and CP. The elevated serum concentration of cytokines involved in inflammatory processes such as IL-6, TGF*β*, and TNF*α* are observed both in PDAC and CP [22, 23]. It has been suggested that stellate cells represent pancreas resident CSC in the course of pancreatic inflammation and can activate the pathways required for malignant transformation of epithelium, promotion of migration, and formation of distant metastases [24].

So far, there is very little data regarding the comparison of the expression of CD24, CD44, and CD133 in PDAC and chronic pancreatitis (CP). There is no sufficient information regarding the role of these biomarkers in CP. However, such comparative analysis might lead to better characterization of stem cell function in both diseases.

## 2. Aim of the Study

The study aimed at examining the expression of CD24, CD44, and CD133 in human PDAC and CP in order to evaluate their clinicopathological correlations and their clinical significance.

## 3. Material

Surgical specimens derived from 23 pancreatic cancer patients (10 women and 13 men, aged 40–75; mean 56.09 ± 9.38) and 15 patients with CP (3 women and 12 men, aged 36–65; mean 48.86 ± 10.57) were subjected to pathology and immunohistochemistry studies. CP and PDAC diagnosis was based on medical history and imaging studies (abdominal ultrasound, EUS, and computed tomography) and confirmed with pathology. Qualification for surgical intervention among CP patients was based on the following indications: detection of tumor in imaging techniques or severe pain unresponsive to medical treatment. The following criteria were used to qualify for resection of PDAC: lack of distant metastases, no infiltration of major blood vessels, and/or lack of invasion of the upper part of the portal vein or the lower part of the superior mesenteric artery that allows for surgical reconstruction of the vessel.

The differentiation grade of PDAC was G1 in 6 cases, G2 in 14 patients, and G3 in 3 individuals. Classification of patients according to the TNM revealed the following stages: pT1 in 3 cases, pT2 in 7 patients, and pT3 in 13 individuals. Lymph node involvement was as follows: N0 in 7 patients, N1a in 5 cases, N1b in 8 patients, and Nx in 3 individuals. Generally, no distant metastases were detected other than small liver metastases (*n* = 2) and splenic vein involvement (*n* = 2).

Parameters related to patient demographics, clinical data, grade, and stage of the disease (according to TNM scale) were correlated with CD24, CD44, and CD133 expression. Patients' survival was calculated from the time of diagnosis until death.

## 4. Methods

Tissue expression of CD133, CD24, and CD44 were assessed with the use of Miltenyi Biotec (Germany), Becton Dickinson (New Jersey, USA), and Dako (Denmark) antibodies (resp.). The intensity and extent of CD24, CD44, and CD133 staining were taken into consideration and scored on a scale of 0–3, in which 0 referred to negative, 1 to weakly positive, 2 to moderately positive, and 3 to strongly positive staining. Seven to ten high-power microscopic fields were evaluated. Staining of KI-67+ cells was evaluated using the computer image analysis system consisting of PC computer equipped with a Pentagram graphic tablet, Indeo Fast card (frame grabber, true color, real time; Taiwan), and Panasonic color TV camera coupled with Carl Zeiss microscope (Germany). This system was programmed by Multiskan 8.08 software, Computer Scanning Systems, Poland. Ki-67 labeling index (LI) was estimated counting 100 cells in ten monitor fields (0.029 mm^2^ each), marking immune-positive cells, so in each case, at least 1000 cells were analyzed. The survival probability of PDAC patients depending on the expression of CD24, CD44, and CD133 was estimated by Kaplan-Meier analysis.

## 5. Results

**Figure 1:**
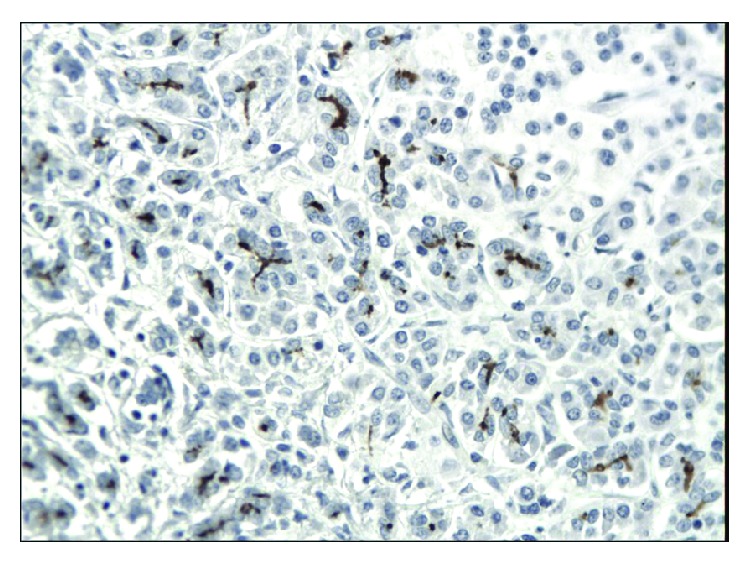
Almost negative immunoexpression of CD24 in epithelial cells in chronic pancreatitis. A weak immunoexpression in stromal cells is seen (magn. 200x).

**Figure 2:**
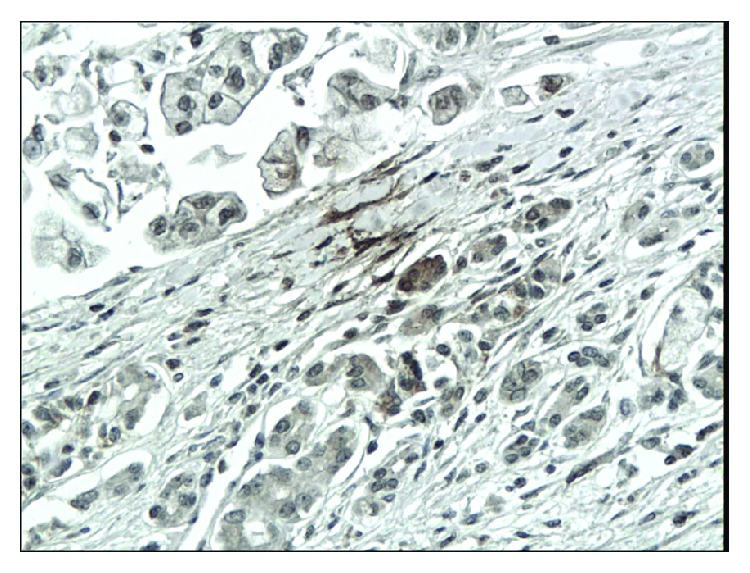
Focal cytoplasmic and membranous immunoexpression of CD24 in pancreatic cancer. A weak immunoexpression of stromal cells is also seen (magn. 200x).

**Figure 3:**
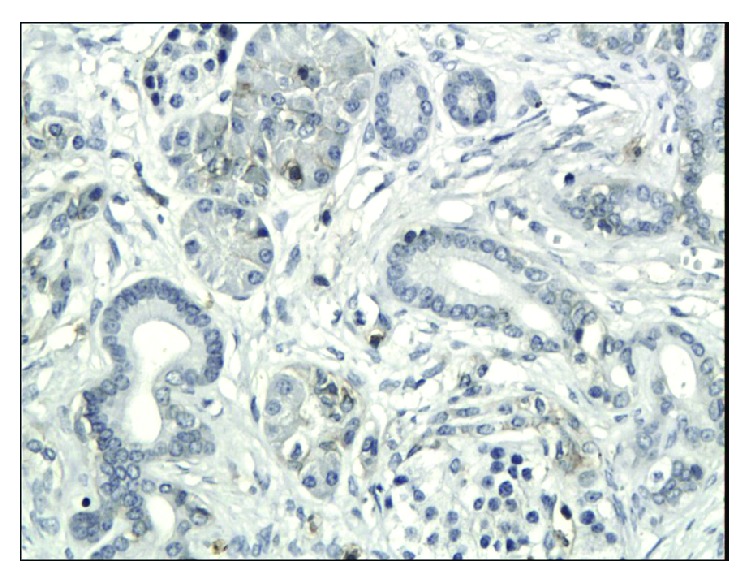
Weak immunoexpression of CD44 in chronic pancreatitis (magn. 200x).

**Figure 4:**
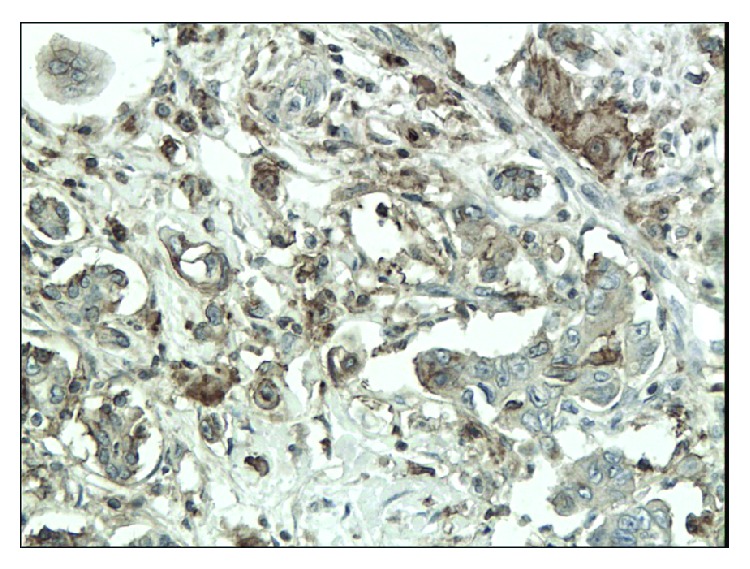
Intense, predominantly cytoplasmic immunoexpression of CD44 in pancreatic cancer (magn. 200x).

**Figure 5:**
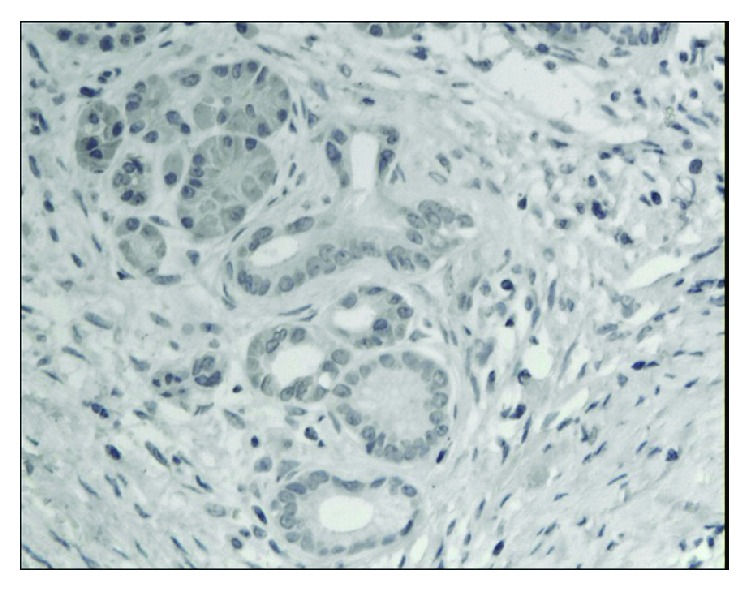
Weak, mainly membranous immunoexpression of CD133 in chronic pancreatitis (magn. 200x).

**Figure 6:**
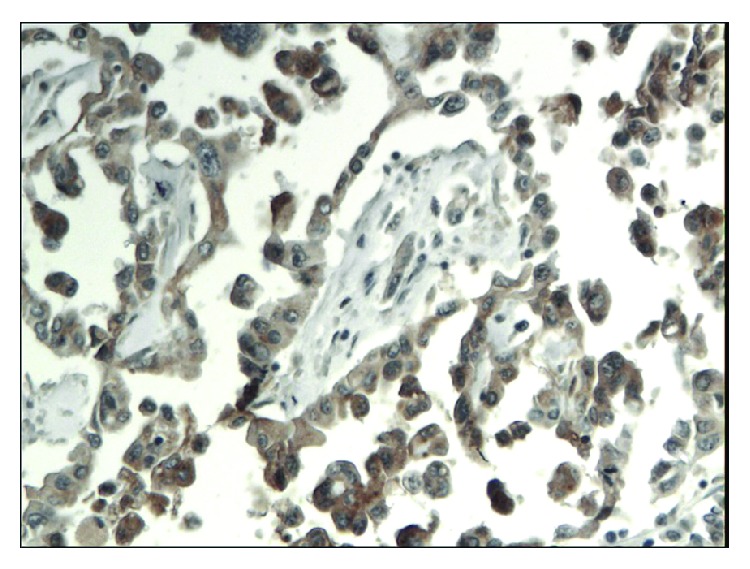
Moderate immunoexpression of CD133 in patients with pancreatic cancer (magn. 200x).

**Figure 7:**
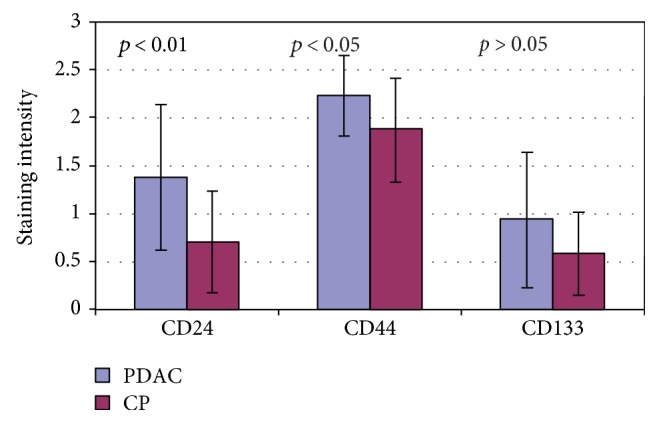
Mean CD24, CD44, and CD133 intensity of staining in PDAC and CP.

**Figure 8:**
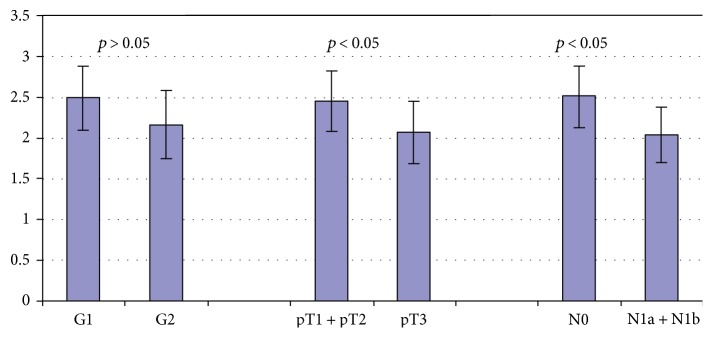
CD44 staining intensity in PDAC depending on tumor differentiation and stage.

**Figure 9:**
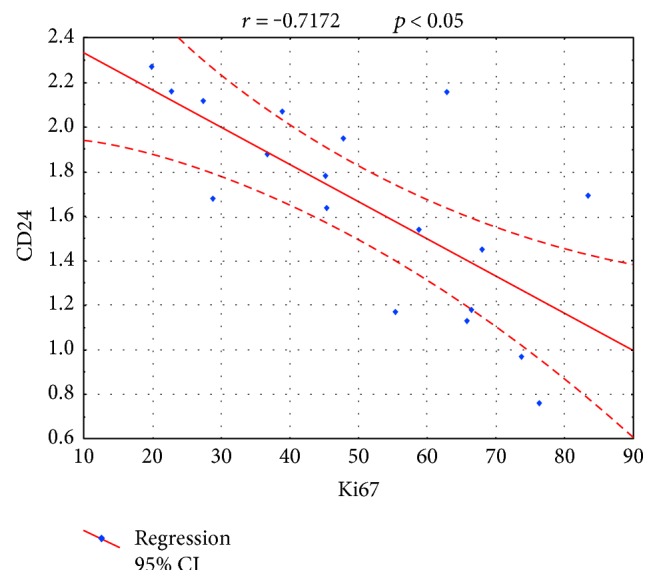
The negative correlation between CD24 staining intensity and Ki67 LI in PDAC.

**Figure 10:**
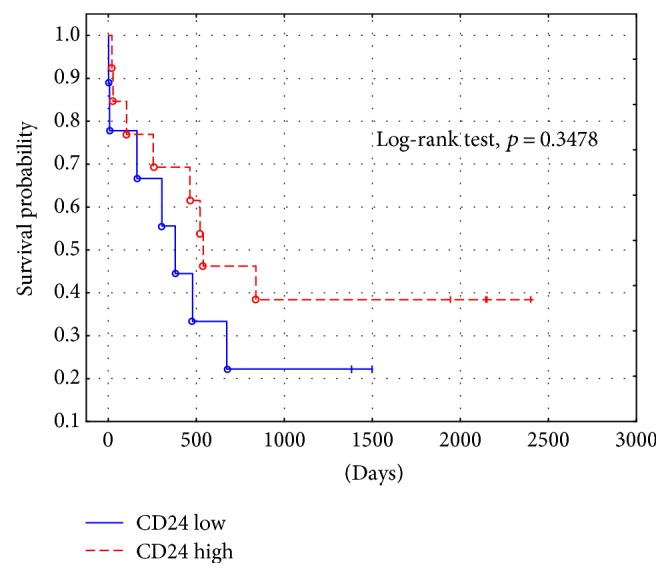
PDAC survival probability depending on CD24 staining intensity.

**Figure 11:**
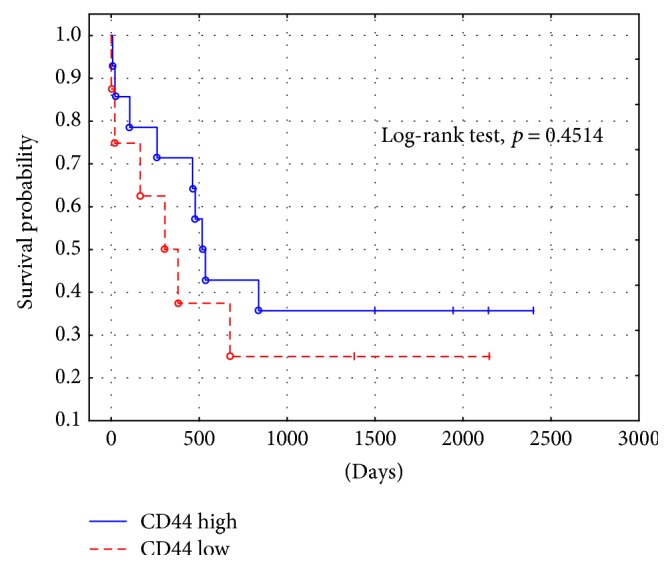
PDAC survival probability depending on CD44 staining intensity.

**Figure 12:**
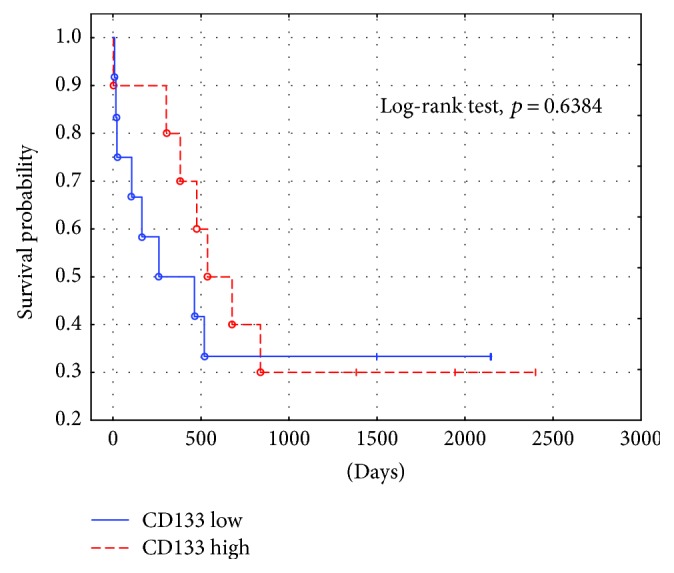
PDAC survival probability depending on CD133 staining intensity.

The immunoexpressions of CD24, CD44, and CD133 were both membranous and cytoplasmic. In PDAC, CD24 immunoreactivity was found in 19 (82.6%) patients, CD133 in 21 (91.3%) and CD44, as well as, Ki-67 in all of examined individuals. In CP, CD24 staining was found in 11 (73.3%) patients, CD133 in 13 (86.6%) and CD44 as well as Ki-67 in all of them. Mean CD24 staining score in PDAC was 1.38 ± 0.76 and was significantly higher than that in CP 0.70 ± 0.53 (*p* < 0.01). In our study, CD44 score in PDAC was 2.23 ± 0.42 and was again significantly higher than that in CP 1.87 ± 0.55 (*p* < 0.05). CD133 score observed in patients with pancreatic cancer was of 0.93 ± 0.58 and was not different from the one detected in CP patients 0.71 ± 0.43 (*p* > 0.05). Both in PDAC and CP individuals, immunohistochemistry demonstrated the lowest expression of CD133 and the highest of CD44 (Figures 1–7). CD44 immunoreactivity was significantly higher (*p* < 0.05) in pT1 and pT2 patients considered together as regards pT3:2.45 ± 0.37 versus 2.06 ± 0.38, as well as in N0 patients compared to those individuals, who presented with involvement of lymph nodes: 2.5 ± 0.38 versus 2.04 ± 0.34 (Figure 8). The immunoreactivity of Ki-67 LI in chronic pancreatitis was higher in PDAC comparing to CP: 54.09 versus 1.56 (*p* < 0.05). We found a negative correlation between the CD24 expression and Ki-67 LI in PDAC (*r* = −0.772; *p* < 0.05) (Figure 9). No significant correlation has been found between CD44 and CD133 expression and Ki-67 LI in PDAC. Additionally, there was no correlation between CD24, CD44, and CD133 expression and patients' age and tumor grade. Moreover, Kaplan-Meier analysis showed no significant relation of survival of PDAC patients and the expression of CD24, CD44, and CD133 (Figures 10–12).

## 6. Discussion

Data regarding the expression of CSC markers, especially those of CD133, CD44, and CD24 in patients with PDAC and/or CP is scarce. Our study showed that the expression of CD133, CD24, and CD44 is present in both CP and PDAC. In addition, the expression of CD24 and CD44 was higher in malignant tumors compared to that in an inflamed pancreatic tissue. No such differences were seen in CD133 expression.

Immervoll et al. analyzed the expression of CD44 and CD133 in surgical samples of PDAC, noncarcinoid pancreatic tumors, and healthy pancreas using immunohistochemistry and immunofluorescence. The authors confirmed that CD44 and CD133 expression is present in normal and inflammatory pancreatic tumors. However, the localization of these markers evaluated by immunofluorescence was different in the pancreatic tissue. In normal pancreas, CD44 and CD133 were located in centroacinar region with CD44 being present in the basolateral membrane and CD133 apically. Interestingly, CD44 and CD133 did not colocalize on the membranes of normal and inflamed pancreatic cells. Similar results were shown in PDAC. No quantitative analysis was done. Additionally, the authors showed that CD44 expression was higher in patients with advanced diseases represented by lymph node involvement (N1) compared to that in individuals without lymph node metastases (N0). The expression of CD44, however, did not correspond to overall survival [25]. In our study, immunohistochemical analysis allowed for detecting only membranous and cytoplasmatic expression of examined biomarkers. The presence of CD44 and CD133 in both PDAC and CP corresponds to our results. We demonstrated higher expression of CD44 in PDAC compared to CP and no significant differences in the expression of CD133 in PDAC and CP. In contrast to the study of Immervoll et al., our data shows the lower immunoreactivity of CD44 in advanced PDAC (T3, N1) in comparison to locally advanced disease (T1, T2, and N0). As shown by many authors, the correlation of CD44 expression and the prognosis in various cancers is not uniform [26–28]. As the role of CD44 in carcinogenesis is not fully elucidated, its prognostic value still cannot be established.

Palagani et al. measured CD44 levels in serum of patients undergoing chemotherapy in PDAC, colon cancer, and gastric cancer. The authors reported a decrease in CD44 levels in patients who responded to treatment [29]. Here, we demonstrated that CD44 expression is higher in patients with pancreatic cancer compared to those with chronic pancreatitis. However, the local advancement of PDAC (T3) and lymph node involvement (N1) inversely correlated with CD44 expression. As CD44 expression has been proved in most malignancies, the exact role of this molecule in carcinogenesis and tumour biology is still unclear. Our results suggest that loss of CD44 expression in advanced PDAC might be a negative prognostic factor in this disease. The molecular background of this phenomenon still needs to be elucidated. Additionally, further studies requiring different analytical methods, such as immunofluorescence, quantitative PCR, or flow cytometry analyses, are needed to confirm this data.

In our study, we have also found similar CD133 immunoreactivity in PDAC and CP patients. Many previous studies confirmed expression of CD133 in majority types of malignant neoplasms, including PDAC [8, 10, 15]. Vizio et al. evaluated the expression of CSC in tissue samples of normal pancreas and PDAC using immunohistochemistry. The authors showed that the immunoactivity of CD133 did not differ between both groups of analyzed patients. Furthermore, CD133 expression did not correlate with tumor stage [30]. Interestingly, similar results were shown in murine model of PDAC. Dosch et al. examined CSC phenotypes in PDAC using lucipherase tags and reported that CD133- and CD24-positive cells were present in both tumorigenic and nontumorigenic cells [31]. Our findings correspond to these observations. As CD133 is an established marker of CSC in PDAC, its expression might not represent disease activity and does not allow for early detection of this neoplasm. One might speculate that the presence of this biomarker in normal or inflamed pancreatic tissue could represent the cells of possible CSC potential, which may be prone to undergo malignant transformation.

Our study also demonstrated that the expression of CD24 was higher in PDAC than in CP. A high expression of this marker was observed in various neoplasms by multiple authors. The meta-analysis presented by Lee et al. analyzed twenty-eight studies on CD24 expression in the breast, female genital tract, gastrointestinal tract, biliary tract, pancreas, urinary system, prostate, and skin carcinomas. Overall, CD24 was more frequently overexpressed in malignant than in benign lesions found within these organs and was significantly associated with lymph node infiltration, advanced clinical stages, and shortened overall survival [32]. Another interesting study on CD24 role in carcinogenesis of PDAC reported increased expression of this marker in preinvasive lesions—pancreatic intraepithelial neoplasia (PanIN). CD24 immunoreactivity was present in 59% specimen of PanIN I, 87% of PanIN II, and 100% of PanIN III [33].

In our study, the values of Ki67 LI in PDAC were higher than those in CP. The expression of Ki67 characterizes the intensity of cell proliferation and is known as a parameter of tumor aggressiveness and prognosis. High Ki67 expression was observed in PDAC by many authors [34–36]. Since CD24 staining intensity was decreasing with higher cell proliferation, it may be possibly considered as an early PDAC marker. Lack of correlation between the expression of CD44 and CD133 with Ki67 immunoreactivity in PDAC might correspond to advanced cell proliferation in all examined specimen including cells without CSC properties. Thus, this finding does not rule out the role of examined CSC biomarkers in the development of PDAC. As to our knowledge, there were no large studies revealing the correlation of CSC markers and Ki67 proliferation index.

Our study showed no correlation between PDAC patients' survival and the expression of CD24, CD44, and CD133. A meta-analysis by Li et al. which analyzed the relation of CD133 expression and survival in PDAC performed on the group of 908 patients showed shorter survival of individuals with high expression of CD133 comparing to those with low expression of this biomarker [37]. Similarly, high expression of CD24 in PDAC correlated with poor prognosis in the study of Lee et al. performed on the group of 67 PDAC with resected tumor [38]. Additionally, an analysis on 96 PDAC patients by Hou et al. showed shorter survival in individuals who presented coexpression of CD44 and CD133 [39]. All presented studies were based only on Asian population, whereas similar data from Western countries is limited. Moreover, all analyses did not provide data on supplemental chemo- or radiotherapy data which also influenced the prognosis of PDAC patients. However, our study analysed a smaller number of patients; it included individuals who did not receive systemic treatment which certainly decreased overall survival rates.

## 7. Conclusions

CD24 and CD44 are upregulated in human pancreatic cancer compared to chronic pancreatitis and may be related to the development of pancreatic cancer. Loss of CD44 expression in advanced stages of PDAC could indicate unfavorable prognosis and may represent a negative prognostic factor. More detailed studies on the role of CD133 cells in both diseases is necessary.
