# Corrigendum to “Expression and Clinical Significance of Cancer Stem Cell Markers CD24, CD44, and CD133 in Pancreatic Ductal Adenocarcinoma and Chronic Pancreatitis”

**DOI:** 10.1155/2017/9617615

**Published:** 2017-08-20

**Authors:** L. Durko, W. Wlodarski, O. Stasikowska-Kanicka, M. Wagrowska-Danilewicz, M. Danilewicz, P. Hogendorf, J. Strzelczyk, E. Malecka-Panas

**Affiliations:** ^1^Department of Digestive Tract Diseases, Medical University of Lodz, Lodz, Poland; ^2^Department of Nephropathology, Medical University of Lodz, Lodz, Poland; ^3^Department of Pathomorphology, Medical University of Lodz, Lodz, Poland; ^4^Department of General and Transplant Surgery, Medical University of Lodz, Lodz, Poland

In the article titled “Expression and Clinical Significance of Cancer Stem Cell Markers CD24, CD44, and CD133 in Pancreatic Ductal Adenocarcinoma and Chronic Pancreatitis” [1], the affiliation listed for Dr. O. Stasikowska-Kanicka and Dr. M. Wagrowska-Danilewicz was incorrect. The corrected authors' list and affiliations are shown above.
